# Human Encephalitis Complicated With Ocular Symptoms Associated With Pseudorabies Virus Infection: A Case Report

**DOI:** 10.3389/fneur.2022.878007

**Published:** 2022-05-09

**Authors:** Liu Yue, Li Yi, Tong Fei, Tian MengWu, Li Man, Wang LiQing, Zou YueLi, Duan JiaLiang, Bu Hui, He JunYing

**Affiliations:** ^1^Department of Neurology, The Second Hospital of Hebei Medical University, Shijiazhuang, China; ^2^Department of Critical Care Medicine, The Second Hospital of Hebei Medical University, Shijiazhuang, China; ^3^Department of Ophthalmology, The Second Hospital of Hebei Medical University, Shijiazhuang, China

**Keywords:** viral encephalitis, next-generation sequencing, severe visual impairment, case report, antiviral therapy

## Abstract

Pseudorabies virus (PRV) is an alpha herpesvirus found in many wild and domestic animals, and causes neurological diseases in humans. Several cases of PRV-induced human encephalitis accompanied with severe visual impairment have been reported. There is currently no effective treatment for severe visual impairment caused by PRV. We report a case of PRV encephalitis with severe visual impairment. The diagnosis and treatment experience of this patient is summarized to improve the awareness of clinicians. We present a 42-year-old man with PRV infection who was admitted due to intermittent fever for 5 days and unconsciousness for 1 day. He subsequently developed severe visual impairment during hospital stay. Empirical antiviral treatment with ganciclovir and sodium foscarnet was started on the day of admission and continued for > 50 days, which had significant treatment effect. Eye complications caused by PRV infection have been frequently reported in patients with PRV encephalitis. In this patient, based on the patient's condition, antiviral therapy was initiated on admission day, and according to the results of the next-generation sequencing of the cerebrospinal fluid, the duration of antiviral therapy was prolonged, which improved treatment efficacy and alleviated neurological symptoms and eye vision damage. To the best of our knowledge, this is the first report that describes partial restoration of acute vision loss associated with PRV infection after aggressive treatment. Our experience suggests that although prompt treatment cannot prevent the acute vision loss associated with PRV infection, timely anti-viral and anti-inflammatory treatment can alleviate ocular complications.

## Introduction

Pseudorabies virus (PRV), namely porcine herpes virus type 1, is a double-stranded DNA virus belonging to the alpha herpesvirus subfamily (1, 2). Since 2018, with the detection of PRV nucleic acids by next-generation sequencing (NGS), several reports from China have shown that PRV can infect humans. In addition, these patients had a very poor prognosis, despite receiving systematic antiviral treatment (3). According to a study, 17.4% (4/23) of patient with PRV infection died, 17.4% (4/23) developed blindness, 21.7% (5/23) patients experienced severe visual impairment, and 65.2% (15/23) patients had severe central nervous system symptoms such as persistent vegetative status, and memory loss (4). In order to improve clinician's understanding of the disease, we report a case of PRV encephalitis with severe visual impairment and analyze and summarize the diagnosis and treatment experience of this patient. To the best of our knowledge, this is the first documented case of PRV infection in which antiviral therapy was started on the day of admission and the duration of therapy prolonged based on NGS results, leading to significant alleviation of encephalitis and ocular symptoms. Here, we report a patient with PRV encephalitis with severe visual impairment who showed significant improvement with anti-viral and anti-inflammatory treatment.

**Figure 1:**
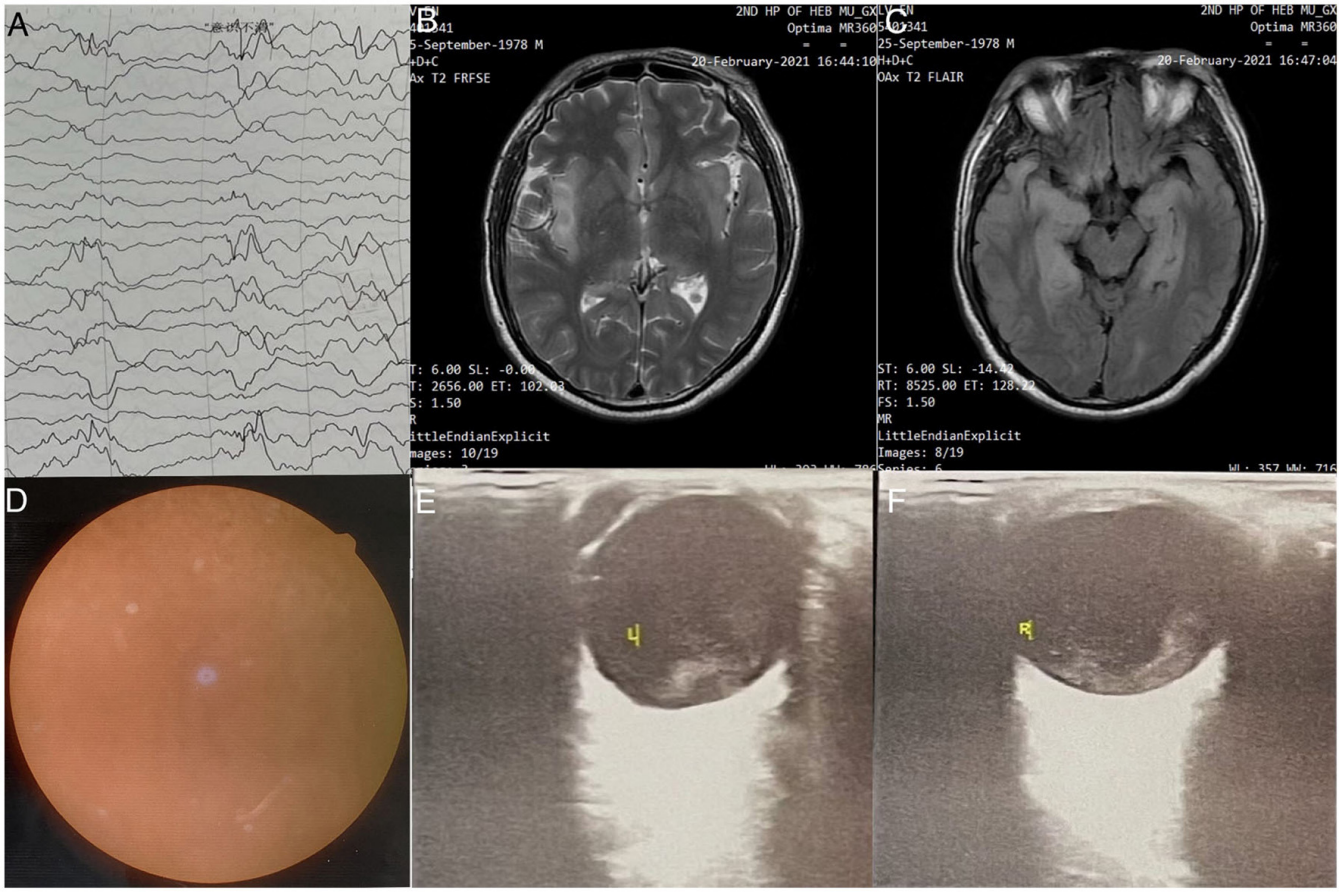
**(A)** Electroencephalography findings: Severe abnormal diffuse mixed slow waves with a lot of fast waves, intermittent low-voltage waves, lasting about 1s, diffuse 1-2Hz irregular s activity, left sharp waves and sharp slow waves are emitted from the front side of the head; **(B)** Head MRI - T2 FRFSE: Bilaterally symmetrical abnormal signals are observed in the anterior cingulate gyrus; **(C)** Head MRI - T2 FLAIR sequence: Symmetrical abnormal signals of insula and fronto temporal lobes; **(D)** Fundus examination: Both eyes have clear cornea with no hyperemia, anterior chambers, pupil diameter of ~3mm, slow reflection of light; **(E)** Ophthalmic ultrasound-L: (1) Severe vitreous opacity; (2) Thickening of the posterior wall of eyeball; (3) Thickening of the four rectus muscles of the eyes; **(F)** Ophthalmic ultrasound-R: (1) Severe vitreous opacity; (2) Thickening of the posterior wall of eyeball; (3) Thickening of the four rectus muscles of the eyes. The ring of the right eye is intact, the lens wave is visible, and the dark area can be seen in the vitreous body. There is medium to high amount of diffuse flocculent weak echo and cluster echo. The posterior wall of the ball is thickened and slightly rough. The thickness of the superior rectus, external rectus, inferior rectus, and medial rectus is ~5.9, 5.6, 5.6, and 6.4mm, respectively.

**Figure 2:**
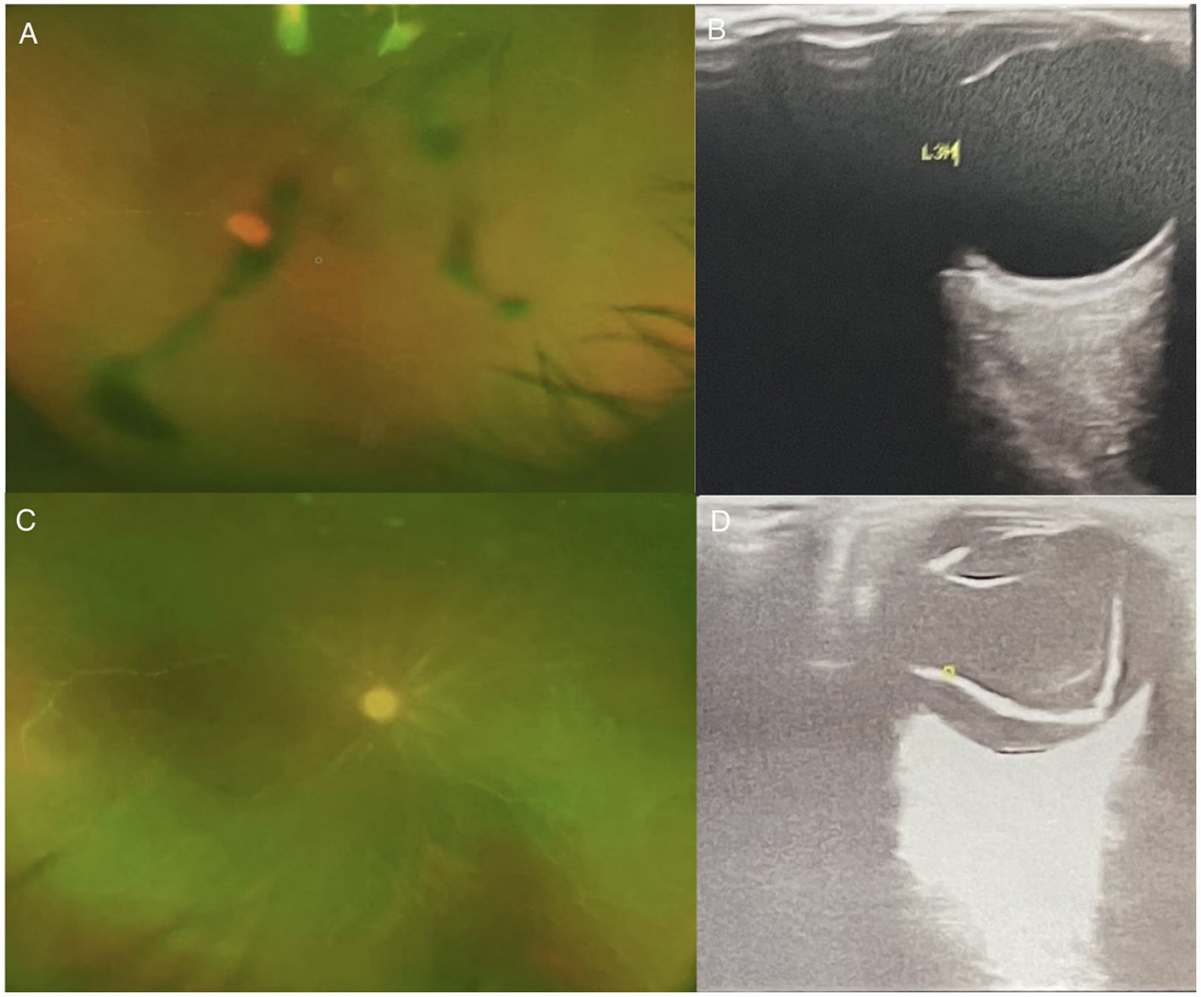
**(A)** Ophthalmology wide-angle lens examination-L; **(B)** Ophthalmic Ultrasound-left: The left eye ring is intact, the lens wave is visible, and the dark area of the vitreous body is moderately diffuse. Spot flocculent weak echo and mass echo, and the posterior wall of the ball is slightly thickened. There is no obvious thickening of the four rectus muscles. **(C)** Ophthalmology wide-angle lens examination-R; **(D)** Ophthalmic Ultrasound-right: The right eye ring is intact, the lens wave is visible, and low and medium amount of diffuse spot flocculent weak echo and cluster echo are seen in the dark area of the vitreous body. Both the horizontal axis and the vertical axis can be detected in the vitreous “shaped echo” band echo. The tip is connected with the optic papilla, and the two ends are connected with the peripheral spherical wall.

**Figure 3:**
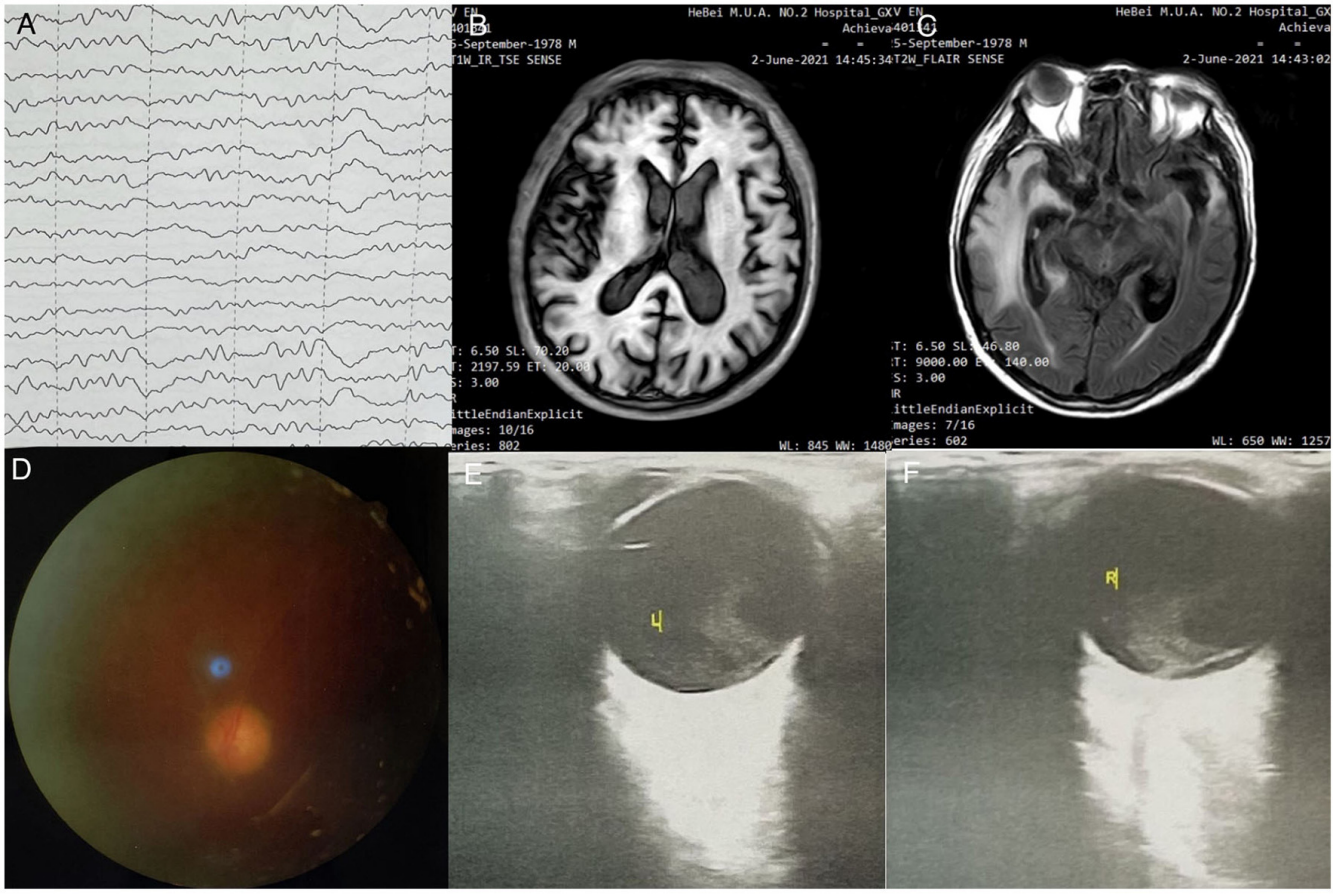
**(A)** EEG examination: moderate to severe abnormal EEG, background diffuse 0, 6 slow wave bursts, 7-8 Hz slow rhythm bursts in the occipital lobe; **(B)** Head MRI - T1IR-TSE: normal head size, bilateral frontal lobe, temporal lobe, insula, bilateral hippocampus, bilateral thalamus, bilateral cingulate gyrus, flaky T1 low signal; **(C)** Head MRI - T2 FLAIR sequence: head size is normal; temporal lobe, insula, and bilateral hippocampus present high signal in T2 FLAIR, high signal in FLAIR; local sulci are clearer than before; **(D)** Fundus examination-left eye; **(E)** Ophthalmic ultrasound-L: severe vitreous opacity, mild thickening of the posterior wall of the eyes; **(F)** Ophthalmic ultrasound-L: severe vitreous opacity, mild thickening of the posterior wall of the eyes, thickening of the inner rectus muscle of the right eye.

A 42-year-old man was admitted to the Second Hospital of Hebei Medical University (Hebei, China), with convulsions for 1 day and headache and fever for 5 days (peak temperature: 39.5°C), accompanied by nausea and vomiting. One day before admission, patient had an episode of altered sensorium and facial twitch. The patient became unresponsive looking at the mobile phone and stopped communicating with his family. Soon, the patient had urinary and stool incontinence. His past medical history was unremarkable. Based on the clinical condition, empirical antiviral therapy were started on the day of admission. Occupational history revealed that his daily work involved close contact with swine and selling pork. Prior to the onset of his symptoms, a large numbers of pigs had died on his farm for no apparent reason. Lumbar puncture (Table 1) was performed and cerebrospinal fluid analysis showed high cerebrospinal pressure with increased white blood cell count, monocyte ratio and absolute monocyte count. NGS of the cerebrospinal fluid detected PRV. Electroencephalography (EEG) showed abnormal findings (Figure 1A). Brain magnetic resonance imaging (MRI) revealed intense T1 and T2 signal changes in the anterior cingulate gyrus, insula, and frontotemporal lobes (Figures 1B,C). On the second day of hospitalization, the patient became comatose and showed signs of respiratory distress with decrease in oxygen saturation. Therefore, he was admitted to the intensive care unit for endotracheal intubation and ventilator-assisted breathing. Ophthalmological examination on 6th day showed no obvious hyperemia in the conjunctiva, but the reflection of light was slow and the fundus was unclear. He developed blurred vision in both eyes gradually, but we did not focus on the condition due to his poor general condition. After treatment for 41 days, NGS of the cerebrospinal fluid detected PRV with 2 unique sequence reads, relative abundance 0.15% and 0.09 % coverage. Forty-one days after admission, the patient complained of lack of light perception (Table 1). Fundus examination showed sluggish light reflex in both eyes (Figure 1D). Ophthalmic ultrasound showed severe vitreous opacity in both eyes (Figures 1E,F). Based on the patient's condition and the results of NGS of the cerebrospinal fluid, antiviral therapy was continued till the NGS of cerebrospinal fluid turned negative (Table 1). Fifty-one days after admission, the patient's family members noticed that the patient was able to see objects within 1 m and could read text (Table 1). On the 57th day after admission, the patient was discharged home for recuperation. Seventeen days after discharge, the patient's level of consciousness was better than that at the time of discharge; however, the patient showed difficulty in communicating with and recognizing people. He was able to walk for a few steps with support. His eyesight visibility was 3.5 m, and he was able to read simple text symbols (Table 1). Follow-up examination at 27 days after discharge showed that the patient's consciousness had improved compared to the last follow-up visit. The patient was still not able to speak fluently and had difficulty in recognizing people. He was able to walk with support. His eyesight visibility was 5 m. Repeat ophthalmological ultrasound showed moderate vitreous opacity in both eyes (more obvious in the left eye), mild thickening of the posterior wall of both eyes, and total retinal detachment in the right eye (Figure 2). Ophthalmology wide-angle lens examination was performed to assess the risk of surgery (Figure 2). Visual acuity examination (left: 0.15, right: 0.1 × 0.5/5 = 0.01) (Figures 3D–F). On follow-up examination 48 days after discharge, the patient was able to walk slowly on his own without support (Figure 3A). The eyesight visibility was 15 m. Repeat head MRI (Figures 3B,C) showed few changes compared with the previous MRI; the local swelling was reduced, and the degree of brain atrophy was aggravated.

**Table 1:** Temporal changes in the findings of cerebrospinal fluid examination and evolution of the patients' ocular involvement during treatment and follow-up.

**Time-point**	**Patient's condition**	**CSF: Routine examination**	**CSF: Biochemistry**	**CSF: Cytology**	**CSF: Next-generation sequencing**
0d	The family members did not report any vision-related problem and the patient was using his mobile phone.				
2d		Cerebrospinal fluid pressure: 210 mmH_2_O Gross appearance: colorless and transparent.	Chlorine 122.6 mmol/L, protein 0.18 g/L, glucose 3.71 mmol/L.	White blood cell count: 25 × 10^6^/L, mononuclear cell ratio: 96%, absolute monocyte value: 2410 × 10^6^/L; protein: negative.	Pseudorabies virus was detected on next-generation sequencing of cerebrospinal fluid. The type was dsDNA, the number of specific sequences was 13996, the relative abundance was 85.79%, and the coverage was 86.02%.
4d		Cerebrospinal fluid pressure: 160 mmH_2_O Gross appearance: colorless and transparent.	Chlorine 128.7 mmol/L, protein 0.14 g/L, glucose 5.26 mmol/L.	White blood cell count: 23 × 10^6^/L, monocyte ratio: 95.7%, absolute monocyte value: 2410 × 10^6^/L, protein: negative.	
6d	The intraocular pressure in both eyes was normal; there was no conjunctival congestion, the cornea was clear, the anterior chamber was normal, the pupil diameter was approximately 3 mm. The patient was in coma.				
41d	Light reflex was sluggish; the fundus of ophthalmoscope was unclear, and the patient complained of lack of light perception. Color ultrasound of the eyes: severe vitreous opacity in both eyes, thickening of the posterior wall of the eyes, and thickening of all four rectus muscles of the eyes.	Cerebrospinal fluid pressure: 120 mm*H*_2_O Gross appearance: colorless and transparent.	Chlorine 109.6 mmol/L, protein 0.58 g/L, glucose 3.32 mmol/L.	White blood cell count: 7 × 10^6^/L, mononuclear cell ratio: 100%, absolute monocyte value: 710 × 10^6^/L; protein: weakly positive.	Pseudorabies virus was detected on next-generation sequencing of cerebrospinal fluid; the type was dsDNA, the number of specific sequences was 2, the relative abundance was 0.15%, and the coverage was 0.09%.
49d	The patient was able to see things.				
51d	The patient's visibility was 1 m and he was able to read text.				
54d		Cerebrospinal fluid pressure: 160 mmH_2_O Gross appearance: colorless and transparent.	Chlorine: 109.6 mmol/L, protein: 0.58 g/L; glucose 3.32 mmol/L.	White blood cell count: 3 × 10^6^/L; protein: weakly positive.	Next-generation sequencing of cerebrospinal fluid showed no clear pathogenic prokaryotic microorganisms, viruses, or eukaryotic microorganisms.
55d	Ophthalmic ultrasound: severe vitreous opacity in both eyes, slight thickening of the posterior wall of both eyes, and thickening of the inner rectus muscle of the right eye.				
73d	The patient's visibility was 3.5 m. He was able to read simple text symbols.				
83d	The patient's visibility was 5 m. Repeat ophthalmological ultrasound: moderate vitreous opacity in both eyes (more obvious in the left eye), mild thickening of the posterior wall of both eyes, and total retinal detachment in the right eye. Improvement in the ophthalmology wide-angle lens examination (picture: J left eye). Visual inspection: the patient's left eye visual acuity was 0.15, and the right eye was 0.1 × 0.5/5=0.01.				
124d	The patient's visibility was 15 m.				

## Discussion and Conclusions

The patient was admitted to the hospital for fever and headache. Based on the clinical experience, empirical antiviral therapy was initiated on the day of admission. However, timely antiviral and immunoglobulin therapy does not seem to stop the progress of panencephalitis. A previous article reviewed 23 cases of encephalitis caused by pseudorabies virus, all of whom had a history of close contact with pigs or pork. In that report, “flu-like” symptoms were observed in the early stage of infection (usually within 7 days), including fever (100%, 23/23), respiratory symptoms (72.7%, 16/22), and headache (57.9%,11/19). Subsequently, these cases showed rapid onset of neurological symptoms, including seizures/convulsions (95.7%, 22/23) and altered consciousness (95.7%, 22/23); 60% (12/20) of the patients showed severe visual impairment, and at the time of publication of this literature review, 65.2% (15/23) of the patients still had severe central nervous system symptoms such as persistent vegetative status, memory loss, and/or ability to only follow simple instructions (4). Lumbar puncture of all cases reviewed showed increased intracranial pressure and lymphocytosis (3–7, 9–11, 14, 15). The present case is in line with the general manifestations of most cases of porcine herpes encephalitis. History of contact with sick pigs is a key pointer toward infection with this pathogen (4). NGS of the cerebrospinal fluid plays an important role in diagnosis. NGS has a high sensitivity and specificity for diagnosis of unknown pathogen infections (3, 16–25). With improvement in the patient's condition, the number of virus copies on NGS gradually decrease, which can facilitate further treatment decision-making (24). Based on the results of NGS, we extended the course of antiviral treatment until the NGS results showed 0 PRV reads in the CSF.

Early and accurate diagnosis of CNS virus infection can help improve the prognosis. Recently, many diagnostic methods for PRV detection have been developed, which can be categorized as serological techniques to detect PRV-specific antibodies and molecular biological methods to detect PRV nucleic acids (16, 25). The Pseudorabies Virus (PRV) g E antibody ELISA detection kit has been widely used, especially in the breeding industry (26).

We reviewed previously published case reports and identified a total of 26 patients with PRV encephalitis. In all 26 patients, the diagnosis was confirmed by metagenomic sequencing of cerebrospinal fluid (Table 2). Plasma antibody test results or cerebrospinal fluid antibody test results were available for 16 patients. Twelve patients (12/16) tested positive for antibodies in plasma or cerebrospinal fluid, 6 patients (6 /16) tested positive for the cerebrospinal fluid antibody, and 5 patients (5/16) tested positive for both plasma and cerebrospinal fluid antibodies. Positive antibody results were measured between days 7 to 46 of disease onset. There were no reports of positive antibody tests in plasma or cerebrospinal fluid within the initial 5 days of disease onset (Table 2). The cerebrospinal fluid NGS test of all 26 patients with PRV encephalitis was found to be positive, and in 9 case reports, the detection time was recorded. All the positive results were tested within 5 days after onset. The detection time of the remaining 17 cases was unknown (Table 2). Based on the literature review, CSF NGS detection seems to be more sensitive than antibody detection in the early stages of PRV encephalitis.

**Table 2:** Summary of human cases of PRV infection reported.

**Case**	**Diagnosis**						**Treatment details**					**Clinical outcomes**	**References**
	**Cerebrospinal** **fluid (NGS)**		**Antibody (ELISA)**				**Ganciclovir**	**foscarnet**	**Acyclovir**	**immunotherapy**	**steroids**		
	**±**	**Onset to**	**CSF+/-**	**Onset to**	**Serum+/-**	**Onset to**	**±**	**±**	**±**	**±**	**±**		
		**sampling**		**sampling**		**sampling**							
1	+	3d	+	23d	+	23d	+	–	+	–	+	The patient can follow instructions, perform eye movements and simple body movements	(5)
2	+	NA	NA	NA	+	>10d	–	–	+	+	+	The patient remained in light coma after 7 weeks of treatment. Consciousness returned at week 12.	(3)
3	+	NA	NA	NA	+	>10d	–	–	+	–	+	The patient was in coma and was kept on a ventilator when he left the hospital, and died after being transferred back to the local hospital.	
4	+	NA	NA	NA	+	>10d	–	–	+	–	+	The patient remained in coma with intermittent convulsions at week 8; slightly improved at week 16.	
5	+	NA	NA	NA	NA	NA	–	–	+	–	+	After one year of follow-up, the patient was blind in both eyes and took care of himself.	
6	+	1d	–	–	+	21d	–	–	+	–	–	1 month after treatment, patient was still dependent on tracheostomy and gastrostomy tube	(6)
7	+	NA	–	–	–	–	+	+	–	–	–	The patient's consciousness and cognitive function improved significantly. At 190-day follow-up, the patient remained blind.	(7)
8	+	1d	+	40d	+	23d	NA	NA	NA	NA	NA	Blindness	(8)
9	+	NA	NA	NA	NA	NA	NA	NA	NA	NA	NA	mild memory impairment	
10	+	NA	NA	NA	NA	NA	NA	NA	NA	NA	NA	Minimally conscious status	
11	+	NA	NA	NA	NA	NA	NA	NA	NA	NA	NA	Persistent vegetative status	
12	+	NA	–	–	gB+	10d	–	–	+	+	+	Patient died after 2 week	(9)
13	+	NA	gE+	10d	–	–	–	+	+	–	+	mRS 3	
14	+	NA	gB+	46d	gE + gB+	46d	–	+	+	+	–	mRS 3	
15	+	NA	–	–	–	–	–	+	+	–	–	Died 2 months later	
16	+	NA	gE + gB+	36d	gE + gB+	36	NA	NA	NA	NA	NA	Died	
17	+	NA	gE + gB+	29d	gE + gB+	10d	NA	NA	NA	NA	NA	Died	
18	+	NA	–	–	gB+	7d	NA	NA	NA	NA	NA	mRS 5	
19	+	3d	NA	NA	NA	NA	–	–	+	+	+	Visual acuity in the right eye of the patient decreased to 2/20.	(10)
20	+	1d	NA	NA	NA	NA	–	+	+	+	+	Slow responses, occasional seizures, mRS	(11)
21	+	2d	NA	NA	NA	NA	–	+	+	+	+	Ventilator-dependent. Follows simple instructions, mRS 3	
22	+	NA	NA	NA	NA	NA	–	+	+	+	+	Follows simple instructions, mRS 3	
23	+	NA	NA	NA	NA	NA	–	+	+	+	+	Ventilator-dependent Slow responses, mRS 3	
24	+	1d	NA	NA	NA	NA	–	+	+	+	+	Blindness, mRs 3	
25	+	5d	NA	NA	NA	NA	+	+	–	–	+	The vitreous opacity of the left eye disappeared, and the occluded retinal vessels remained unchanged.	(12)
26	+	2d	–	2d	–	2d	–	+	+	+	+	The patient's prognosis is very poor, and mechanical ventilation is still required.	(13)

In our patient, there were no ophthalmological symptoms at the onset. The patient quickly developed altered sensorium. Forty-one days after admission, the patient showed recovery of disturbed consciousness and complained of lack of light perception. According to recent reports, it is not uncommon for PRV viral encephalitis to cause bilateral necrotizing retinitis (3, 11, 15). In the review of 23 cases of encephalitis caused by PRV, 17.4% (4/23) developed blindness, and 21.7% (5/23) patients experienced severe visual impairment (4). Visual impairment can appear in the early stage or in the late stage (3, 11, 15, 24). Close attention should be paid to visual impairment in these patients, as the symptoms may be masked by altered consciousness. At present, pars plana vitrectomy and silicone oil injection are the main treatment measures for the ocular complications of PRV such as retinal detachment, acute retinal necrosis syndrome, and severe visual impairment (24, 25). Despite active treatment, the prognosis of patients with the ocular complications of PRV is poor.

A total of 19 cases of PRV encephalitis patients were retrieved for the antiviral treatment regimens after evaluation of the case reports (Table 2). Seven patients were treated with acyclovir alone, 9 patients were treated with a combination of acyclovir and potassium phosphate, and 2 patients were treated with ganciclovir; none of the cases was treated with sodium phosphate alone. Moreover, 1 patient was prescribed empirical treatment with acyclovir, which was subsequently changed to ganciclovir after definitive diagnosis (Table 2). Among the reviewed case reports, two reports described treatment with a combination of ganciclovir and foscarnet. In the first case, the consciousness and cognitive function of the patient with PRV encephalitis were significantly improved (7). Another patient had decreased visual acuity in both eyes and disappeared vitreous opacity in the left eye (12). By reviewing the 19 cases, it is difficult to compare the efficacy of the different antiviral regimens. Fan S. reported treatment of 17 patients with PRV encephalitis with acyclovir monotherapy, 4 of whom died, and the remaining patients had severe residual neurological deficit (9). In one particular case, after 24 days of acyclovir combined with foscarnet, the patient was still in deep coma and on mechanical ventilation (13). The above reports suggest that PRV encephalitis may not respond well to acyclovir. Presently, there is no relevant controlled drug trial and the effectiveness of antiviral drugs for PRV encephalitis still needs to be evaluated further.

Based on our literature review, a total of 19 patients who received specific treatment regimens were identified. Six PRV patients (6/19) were treated with glucocorticoids alone, 10 patients (10/19) received intravenous immunoglobulin (IVIg) in combination with glucocorticoids, while 3 PRV patients (3/19) were not treated with intravenous immunoglobulin (IVIg) or glucocorticoids (Table 2). According to one article, once a PRV patient is diagnosed, treatment should be started immediately, including human immunoglobulin, glucocorticoids, antiviral drugs, along with symptomatic and supportive treatments. In severe cases, intravenous immunoglobulin and glucocorticoid therapy can save the life of the patient (27, 28). A study suggested that severe visual impairment can be avoided with antiviral and corticosteroid therapy (11). Immunotherapy was used in most previously reported cases. Nonetheless, there is no relevant controlled drug trial. Thus, there is a lack of robust evidence of the effectiveness of human immunoglobulin and glucocorticoids in PRV patients.

In this case, we initiated the empirical antiviral therapy on the day of admission. Subsequently, based on the patient's condition and the result of NGS, antiviral therapy was continued to more than 50 days until the NGS results showed 0 PRV reads in the CSF. The consciousness, motor function and vision of the patient were improved gradually. On day 48 after discharge, the patient was able to walk slowly on his own without support and his visibility was 15 m. Forty-eight days after discharge, the patient was able to walk slowly on his own without support and his visibility was 15 m. In this case, early and long-term antiviral therapy and early immuno therapy may be the reasons for the better curative effect. In this case, the patient's visual function improved but retinal detachment occurred in the right eye later. This suggests that although the vision loss in the acute phase can improve with antiviral and anti-inflammatory treatment, the risk of subsequent retinal peeling cannot be ignored. Therefore, during follow-up, regular fundal examination should be conducted to monitor for potential retinal detachment. Our case may provide new insights for the treatment of patients with PRV encephalitis and can help reduce the disability rate and improve the quality of life of patients to a certain extent.

## Data Availability Statement

The original contributions presented in the study are included in the article/supplementary material, further inquiries can be directed to the corresponding authors.

## Ethics Statement

Written informed consent was obtained from the individual(s) for the publication of any potentially identifiable images or data included in this article.

## Author Contributions

LYu: data collection and analysis, manuscript writing, and literature research. LYi: decision making of patient's diagnosis and treatment, contributed ideas to the article, and supervision of the work. LM and WL: patient follow-up. ZY: cytological examination and cerebrospinal fluid examination. DJ: assessment of the patient's ophthalmic condition. BH and HJ: diagnosis and treatment experience sharing and guidance. TF and TM: treatment of patients in the ICU. All authors contributed to the article and approved the submitted version.

## Funding

This article was supported by the grants from the Natural Science Foundation of Hebei Province (No. H2021206461) and the National Natural Science Foundation of China (No. 81701264).

## Conflict of Interest

The authors declare that the research was conducted in the absence of any commercial or financial relationships that could be construed as a potential conflict of interest.

## Publisher's Note

All claims expressed in this article are solely those of the authors and do not necessarily represent those of their affiliated organizations, or those of the publisher, the editors and the reviewers. Any product that may be evaluated in this article, or claim that may be made by its manufacturer, is not guaranteed or endorsed by the publisher.

## Abbreviations

CSF: Cerebrospinal fluid
PRV: Pseudorabies virus
LS: Lumbosacral
T: Thoracic
L: Lumbar
